# Dynamic Expression and Gene Regulation of MicroRNAs During Bighead Carp (*Hypophthalmichthys nobilis*) Early Development

**DOI:** 10.3389/fgene.2021.821403

**Published:** 2022-01-19

**Authors:** Jianjun Fu, Wenbin Zhu, Lanmei Wang, Mingkun Luo, Bingjie Jiang, Zaijie Dong

**Affiliations:** ^1^ Key Laboratory of Freshwater Fisheries and Germplasm Resources Utilization, Ministry of Agriculture and Rural Affairs, Freshwater Fisheries Research Center of Chinese Academy of Fishery Sciences, Wuxi, China; ^2^ Key Laboratory of Exploration and Utilization of Aquatic Genetic Resources, Ministry of Education, Shanghai Ocean University, Shanghai, China; ^3^ Wuxi Fisheries College, Nanjing Agricultural University, Wuxi, China

**Keywords:** bighead carp, embryo, larva, development, microRNA, dynamic regulation

## Abstract

The early development of fish is regulated through dynamic and complex mechanisms involving the regulation of various genes. Many genes are subjected to post-transcriptional regulation by microRNAs (miRNAs). In the Chinese aquaculture industry, the native species bighead carp (*Hypophthalmichthys nobilis*) is important. However, the genetic regulation related to the early development of bighead carp is unknown. Here, we generated developmental profiles by miRNA sequencing to study the dynamic regulation of miRNAs during bighead carp early development. This study identified 1 046 miRNAs, comprising 312 known miRNAs and 734 uncharacterized miRNAs. Changes in miRNA expression were identified in the six early development stages. An obviously increased expression trend was detected during the development process, with the main burst of activity occurring after the earliest stage (early blastula, DS1). Investigations revealed that several miRNAs were dominantly expressed during the development process, especially in the later stages (e.g., miR-10b-5p, miR-21, miR-92a-3p, miR-206-3p, and miR-430a-3p), suggesting that these miRNAs exerted important functions during embryonic development. The differentially expressed miRNAs (DEMs) and time-serial analysis (profiles) of DEMs were analyzed. A total of 372 miRNAs were identified as DEMs (fold-change >2, and false discovery rate <0.05), and three expression profiles of the DEMs were detected to have co-expression patterns (*r* > 0.7, and *p* < 0.05). The broad negative regulation of target genes by miRNAs was speculated, and many development-related biological processes and pathways were enriched for the targets of the DEMs, which might be associated with maternal genome degradation and embryogenesis processes. In conclusion, we revealed the repertoire of miRNAs that are active during early development of bighead carp. These findings will increase our understanding of the regulatory mechanisms of early development of fish.

## Introduction

The early development of animal is a complex process, characterized by spatiotemporal changes in gene expression and morphology (Aanes et al., 2014). Advances in next-generation sequencing (NGS) technologies have led to progress in the field of gene expression and its regulation mechanisms in the early development of fish. The dynamic and stage-specific gene expression characteristics (or profiles) of the early stages of development have been demonstrated widely in studies of fish life-history (Sarropoulou et al., 2016; Rauwerda et al., 2017; Fu et al., 2019). Meanwhile, many new insights into gene-expression regulation during animal development have been obtained, including transcription factors and non-coding regulatory RNAs (Nepal et al., 2013; Gaitán-Espitia and Hofmann, 2017; Yan et al., 2020). MicroRNAs (miRNAs) comprise an evolutionarily conserved category of small regulatory RNAs (∼22 nt), which mainly inhibit mRNA translation to regulate gene expression at the post-transcriptional level (Gavery and Roberts, 2017). Whereas, many species- or group-specific miRNAs that act in the establishment of evolutionary novelties (Chen and Rajewsky, 2007; Wu et al., 2009; Lee et al., 2010). Several studies have characterized miRNAs’ functional importance in diverse biological processes, such as growth (Yi et al., 2014), tissue regeneration (Rajaram et al., 2014), environmental stress (Sun et al., 2020), and immune responses (Qiao et al., 2021). To date, several studies have focused on the miRNAs’ roles of gene regulation during animal development (Alberti and Cochella, 2017; Chen et al., 2020; Wu et al., 2020; Jing et al., 2021; Kusama et al., 2021). In teleosts, the dynamics of gene expression and its regulation in early development have been described in model fishes, such as zebrafish (*Danio rerio*) (Nepal et al., 2016; Kasper et al., 2017; Shi et al., 2020) and medaka (*Oryzias latipes*) (Tani et al., 2010), and important aquaculture species (Rasal et al., 2016; Herkenhoff et al., 2018), such as rainbow trout (*Oncorhynchus mykiss*) (Ramachandra et al., 2008), turbot (*Scophthalmus maximus*) (Robledo et al., 2017), Nile tilapia (*Oreochromis niloticus*) (Pinhal et al., 2018), European sea bass (*Dicentrarchus labrax*) (Sarropoulou et al., 2019), and the common carp (*Cyprinus carpio*) (Wang et al., 2017; Wang et al., 2021).

The eastern Asian native species, bighead carp (*Hypophthalmichthys nobilis*), has been introduced widely into nonnative waters, and has become a major aquaculture species in China. The production of bighead carp was 3.14 million tons in 2018, accounting for 5.8% of the worldwide freshwater finfish production (FAO, 2020). However, in past decades, because of overfishing, pollution, habitat destruction in natural waters, and inbreeding occurring in hatcheries with successive artificial breeding practices for more than 60 years, the fishery resources and seed quality of bighead carp have declined (Chen et al., 2012; Hu et al., 2015). Specifically, phenotypic abnormalities and high mortality in early development were detected in hatcheries. Meanwhile, a decline in growth performance is commonly found in commercial husbandry. In contrast, the bighead carp is considered an invasive species in some countries, such as the United States, where it has had a detrimental environmental impact because of its rapid reproduction in nonnative waters (Lamer et al., 2015; Farrington et al., 2017). Early development is often remarkably resilient but is also adaptable in response to environment changes, representing the most important developmental period leading to high performance and superior quality in subsequent developmental phases (Garfield et al., 2013; Pittman et al., 2013). For example, the early development of bighead carp is implement well within the optimal temperature (approximately ranged from 22 to 26°C), and present with diversity in hatching duration under different temperatures. However, hatching with lower or higher temperature generally cause higher mortality and abnormality of embryos and larvae, which leading to low quality of juveniles and loss in yield. Therefore, investigating the genetics of early development in the bighead carp will provide a basis for genetic knowledge of the species, and might contribute to a better understanding of early life processes and their fitness to the environment.

The phenotypic changes in embryogenesis and larval development of bighead carp have been well described (Yi et al., 1988) and analyzed under different conditions (Gerorge and Chapman, 2013). Recently, a study revealed the dynamics of gene expression, and many differentially expressed genes were identified that are related to early development events, e.g., maternal to zygotic transition (MZT) and hatching (Fu et al., 2019). However, gene expression regulation during early development of bighead carp has received little attention and genetic information is lacking. To better understand the regulation of bighead carp early development, a high-throughput sequencing strategy was used in the present study to determine expression levels of miRNAs during the six early development stages in bighead carp (i.e., early blastula, six-somite embryo, 26-somite embryo, hatching stage, 12 h post-hatching (hph) larva, and 24 hph larva). Pairwise differential expression between adjacent stages, and the expression profiles of miRNAs throughout the experimental period were analyzed. Putative targets of the miRNAs were predicted and their expression levels were verified, and the significantly negative regulated genes (or transcripts) were subjected to further analyses. Enrichment analyses were carried out for miRNAs’ functional evaluation, based on target annotations in the Gene Ontology (GO) and Kyoto Encyclopedia of Genes and Genomes (KEGG) databases. Ten differentially expressed miRNAs (DEMs) and all five targets of them were validated using qRT-PCR. The high coincidence was detected between the RNA-sequencing (RNA-seq) and qRT-PCR data. The results of this study, not only provided a valuable genetic basis to understand the dynamics and regulation of miRNAs during bighead carp early development, but also expanded the miRNA resources in teleost species.

## Materials and Methods

### Experiment and Sampling

The mass spawning of parental fish and hatching of fertilized eggs were carried out at the Suzhou Wei-Lai Aquatic Breeding Farm (Provincial hatchery for Chinese carps, Jiangsu, China), the details were the same as those of a previous study (Fu et al., 2019).

The development stages of the embryos were observed under a microscope. The samples were collected at the following six developmental stages: Early blastula (developmental stage (DS) 1; approximately 3.5–5 h post-fertilization, hpf), the six-somite embryo (DS2, approximately 15–16 hpf), the 26-somite embryo (DS3, approximately 23–24 hpf), the hatching stage (DS4, approximately 29–30 hpf), 12 hph larva (DS5), and 24 hph larva (DS6). For the convenience of analysis, a total of eighteen samples were collected from the previous study (Fu et al., 2019), consisting of three replicates at each stage, whereas five replicates for each stage were used previously. In detail, fifteen embryos (DS1, DS2, and DS3) or ten larvae (DS4, DS5, DS6) were pooled together for each sample. All samples were washed rapidly using phosphate-buffered saline (PBS) and then stored in the RNA store Reagent (Tiangen, Beijing, China) at −80°C for further use.

### Extraction of RNA, Construction of the Libraries, and Sequencing

A mirVana miRNA Isolation Kit (Ambion, Austin, TX, United States) was used to extract the total RNA from the samples according the supplier’s instructions. DNAse I (New England Biolabs, Ipswich, MA, United States) was used to remove genomic DNA contamination from the RNA samples. A NanoDrop 2000c spectrophotometer (Thermo Fisher Scientific, Waltham, MA, United States), 1.5% agarose gel electrophoresis, and the Agilent Bioanalyzer 2,100 system (Agilent Technologies, Santa Clara, CA, United States) were used to evaluate the RNA content and purity. The samples with A260/280 ranged from 1.8 to 2.1 and the RNA Integrity Number (RIN) ≥ 7 were retained for subsequent analysis.

All eighteen samples were prepared and used for library construction, following the instructions provided by the manufacturer. Briefly, ligated with 3′ and 5’ adapters (Illumina, San Diego, CA, United States) using T4 ligase (New England Biolabs, Ipswich, MA, United States), purified RNA was reverse-transcribed into the first strand cDNA and amplified by PCR using primers complementary to the adaptor sequences. The final small RNA library was prepared by purified the nucleotide fractions at 145–160 nt length. And then, the libraries were subjected to single-end sequencing using the Illumina Hiseq X Ten platform and 150 bp single-end sequencing technology (Illumina, San Diego, United States) at the OE biotech company (Shanghai, China). All sequencing reads were exported in the FASTQ format.

### Sequence Reads Analysis and Identification of miRNAs

FastQC v0.11.2 (http://www.bioinformatics.babraham.ac.uk/projects/fastqc/) was used to assess for quality control of the raw sequence data. The adapters were clipped using cutadapt (Martin, 2011). FASTX-Toolkit (http://hannonlab.cshl.edu/fastx_toolkit) was used to trim low-quality reads (Q20 < 80%). NGSQCToolkit (Patel and Jain, 2012) was used to remove the reads with unknown bases (“N”). Finally, the reads ranged from 15 nt to 41 nt were reserved for further study. The SOAP program (http://soap.genomics.org.cn) was used to map the clean reads to the reference genome with a tolerance of one mismatch. The non-coding RNAs were annotated as small nucleolar RNAs (snoRNAs), small nuclear RNAs (snRNAs), tRNAs, and rRNAs, which were then aligned and subjected to BLAST searching against the GenBank (http://www.ncbi.nl.nih.gov/genbank/) and Rfam v.14.6 (http://rfam.xfam.org/) databases. Alignment at the miRBase v.22.1 database (http://www.mirbase.org/) identified the known miRNAs. The algorithm miRDeep 2 (Friedländer et al., 2012) was used to analyze those miRNAs that showed no similarity to conserved or existing miRNAs, thus identifying them as possible uncharacterized miRNAs. The corresponding miRNA star sequence was also identified using the pre-miRNA hairpin structure and the miRBase database. The length distribution of small RNAs was visualized using ggplot2 package (part of the R project (http://www.R-project.org)).

### Differential miRNA and Expression Profiles Analyses

The read count frequency of the miRNAs was normalized using transcripts per million (TPM). Next, the R project packages Hmisc, reshape2, dplyr, ggplot2, and UpSetR were used to analyze the statistics for the TPM values of the miRNAs, for hierarchical clustering (Pearson’s correlation), for principal component analysis (PCA) for the eighteen samples, and to identify the intersecting sets of extremely highly expressed miRNAs (TPM ≥1,000), respectively.

Differentially expressed miRNAs (DEMs) between adjacent stages were identified based on the read counts of miRNAs using the DESeq2 package (Love et al., 2014) in the R project. The miRNAs were considered significant DEMs only if the adjust *p*-value (false discovery rate, FDR) below 0.05 and | log_2_ (fold change, FC) | >1 (or 2). An online resource (http://bioinformatics.psb.ugent.be/webtools/Venn/) was used to depict the Venn diagram of pairwise DEMs. The STEM software (Ernst and Bar-Joseph, 2006) was used for expression profile analysis according to the DEMs’ mean TPM values at each stage (mean TPM ≥1 at least in one stage), using default parameters, i.e., minimum correlation of 0.7 and a significance threshold of 0.05 (Bonferroni method).

### Prediction of Targets of miRNAs and Their Functional Enrichment

To determine the DEMs’ functions, the target mRNAs of the DEMs were predicted using miRanda software (Enright et al., 2003), with the parameters: S ≥ 150, ΔG ≤ −30 kcal/mol, and with strict 5′ seed pairing. Pearson’s correlation test was carried out to identify miRNA-mRNA pairs that correlated negatively, based on miRNA normalized data (TPM) and mRNA normalized data [Fragments Per Kilobase of transcript per Million mapped reads (FPKM)] from a previously study (Fu et al., 2019), using the Hmisc package in the R project. Target mRNAs that presented significantly negative correlation values (*p* < 0.05) with the miRNAs were chosen for further analyses. Cytoscape v3.6.1 software (Shannon et al., 2003) was used to visualize the miRNA-mRNA regulatory networks.

Based on the corresponding targets of the DEMs, GO (http://www.geneontology.org) biological process categories, and KEGG (http://www.genome.jp/kegg/pathway.html) pathways were analyzed using the clusterProfiler package (Wu et al., 2021) for functional enrichments; terms with *p* < 0.05 were defined as significantly enriched. The enrichment results were visualized using the ggplot2 package in the R project.

### Quantitative Real-Time Reverse Transcription PCR Assay

Total RNAs were reverse transcribed to cDNA using a PrimeScript RT Reagent Kit (Takara Bio, Dalian, China). The expression levels of 10 DEMs (DS2 *vs* DS1) were then assessed using qPCR on a CFX-96 Real-time PCR System (Bio-Rad, Hercules, CA, United States) in 20 μl reaction volume, comprising 10 μL of SYBR Premix kit 2× (Takara Bio), 0.5 μl of the miRNA-specific forward primer (10 μM) (Supplementary Data Sheet S1), 0.5 μl the miScript universal primer (10 μM), and 2 μl the cDNA prepared above. The reaction conditions comprised: initial denaturation at 95°C for 10 s; followed by 40 cycles of 95°C for 5 s, 60°C for 15 s, and a final cycle at 95°C–65°C. In addition, five target transcripts were assessed using the qPCR method according to the previous study (Fu et al., 2019), primer pairs provided in the Supplementary Data Sheet S1. Triplicate reactions were conducted, and the relative expression levels of the miRNAs and targets were normalized against the expression of the *5s rRNA* and *actb* gene using the 2^-∆∆Ct^ method (Livak and Schmittgen, 2001), respectively.

The F-Test and Shapiro-Wilk test were performed to test for homogeneity and normality, respectively, followed by a one-tailed Wilcoxon test and one-tailed T-Test for differential expression (DS2 *vs* DS1) analysis, which were carried out using Excel (Microsoft, Redmond, WA, United States) and the R project package stats. Pearson’s correlation analysis and heatmap construction of relative miRNA expression (qRT-PCR data) and the TPM values were carried out using the R project packages Hmisc and pheatmap, respectively.

## Results

### Overview of the RNA-Sequencing

A total of 818, 358, 688 raw reads, with a mean of 45, 464, 372 raw reads from each library were obtained (Supplementary Data Sheet S2). After filtering out low-quality reads, and removing adaptor sequences, the clean reads were retrieved for further analyses. The sequencing data were deposited in the NCBI Sequence Read Archive (SRA) repository with the accession number: PRJNA781113.

The clean reads’ length distribution is shown in Supplementary Figure S1. The later five stages (DS2, DS3, DS4, DS5, and DS6) were basically the same, with a single peak at 22 nt, whereas the DS1 stage had two peaks at 22 nt and 28 nt. The concentrations of 21–23 nt sequences showed a gradually increasing trend during the development process, i.e. from 18.74% in DS1 to 65.47% in DS6. Whereas, the 26–28 nt fraction showed a gradually decreasing trend during the development process, i.e. from 6.08% in DS1 to 1.20% in DS6 (Supplementary Data Sheet S2).

### Statistics of miRNA Identification and Predictions

**FIGURE 1:**
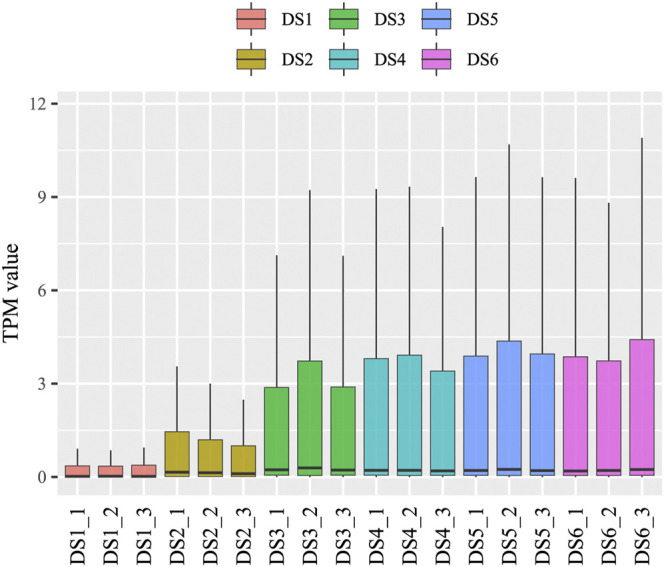
A boxplot of miRNA expression for each sample.

**FIGURE 2:**
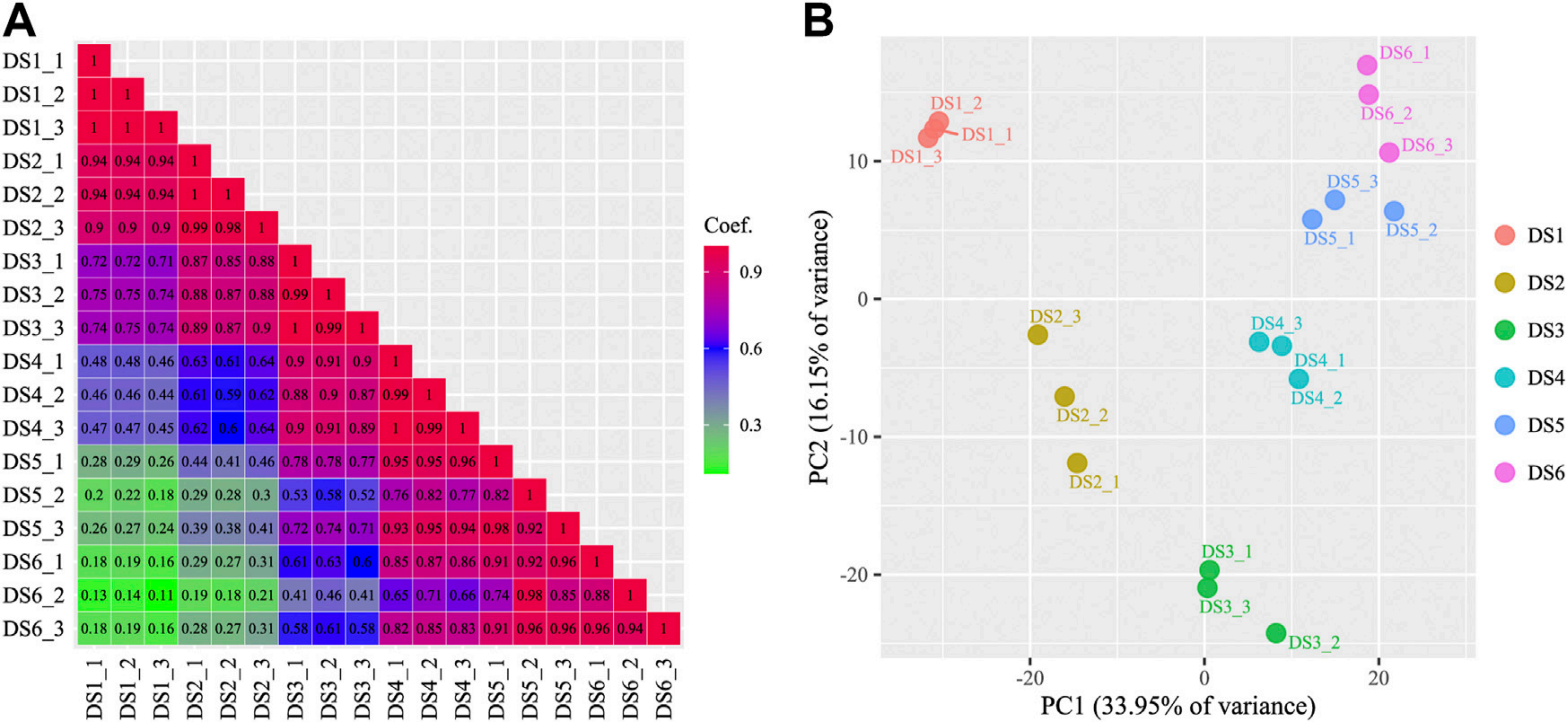
A matrix showing the correlations among samples **(A)** and a scatter plot of samples based on principal component analysis **(B)**.

An increasing trend of known miRNA counts was detected in the clean reads during the development process. Similar categories of known and uncharacterized miRNAs were detected in the later five development stages (734–850), whereas in the earliest stage (DS1), slightly fewer miRNA categories were detected (575–594) (Supplementary Data Sheet S3). The present study identified 312 known miRNAs and 734 uncharacterized miRNAs, which belong to 109 miRNA families. The read counts and TPM values were measured for each miRNA in each sample (Supplementary Data Sheet S4), and used for subsequent analyses. A broad range of read counts was detected both for known miRNAs (1–2,316,158) and uncharacterized miRNAs (1–13,440). A boxplot of miRNA abundance for the eighteen samples is shown in Figure 1, which shows an obviously increasing trend during the earlier development stages (DS1, DS2, and DS3). Highly consistency was detected among replicates from each development stage (*r* ≥ 0.82, *p* < 0.01) (Figure 2A), which corresponded to the scatter plot based on the PCA data (Figure 2B). Furthermore, the PCA data showed clear separation of the earlier stages from the later stages, especially for the first sampled stage (DS1).

At the global level, the known miRNAs had higher expression levels than the uncharacterized miRNAs during all the sampled stages (Supplementary Figure S2A). In detail, the intersections of 52 extremely highly expressed miRNAs (TPM ≥1,000) in the six stages were analyzed (e.g., miR-1, miR-9-4-3p, miR-9-5p, miR-20a-5p, miR-20b-5p, miR-21, etc., Supplementary Data Sheet S4), and an increasing trend of counts was detected during the development process, i.e., from 3 in DS1 to 49 in DS6 (Supplementary Figure S2B). Interestingly, all those items were known miRNAs, even in the secondary expression level (TPM <1,000, and ≥100), only one uncharacterized miRNA (novel276_star) were detected among 80 miRNAs (Supplementary Data Sheet S4).

To investigate the important miRNAs in early development, the ten most abundant miRNAs (according to the TPM) for each development stage were identified, and which comprised seventeen known miRNAs (Table 1), five of which (miR-21, miR-10b-5p, miR-92a-3p, miR-206-3p, and miR-430b-3p) were the top miRNAs at least in five stages. In addition, the ten miRNA families with highest mean TPM values were as follows: miR-1, miR-9, miR-10, miR-17, miR-21, miR-22, miR-26, miR-203, miR-363, and miR-430 (Supplementary Data Sheet S4).

**TABLE 1:** The most abundant microRNAs (highlighted) for each stage and their target genes in bighead carp.

miRNA	DS1	DS2	DS3	DS4	DS5	DS6	Target gene
miR-1	371	334	4,516	15,860	29,629	37,759	—
miR-9-5p	380	615	2,895	8,703	32,290	57,698	—
miR-10a-5p	174	3,815	9,535	8,446	9,372	9,136	*cyb5r4*; *ipo13*; *pik3r4*
miR-10b-5p	397	7,134	21,934	22,557	26,853	25,605	*pik3r4*
miR-10d-5p	150	1,469	6,878	7,199	10,705	12,061	—
miR-21	324	8,172	15,376	13,957	13,822	16,713	*ipo4*
miR-22a-3p	455	1,644	6,406	10,161	14,212	13,360	—
miR-92a-3p	340	10,678	16,984	11,574	10,886	9,631	*rbp4*
miR-100-5p	150	337	3,486	7,185	12,226	13,288	*abcc8*; *tmem47*; *cntnap2*
miR-199-5p	123	298	2,734	6,199	10,439	11,442	*mxra8*; *podxl*; *gatad2b*; *alpk3*
							*kbtbd2*; *phka*; *ddx56*; *eps15l1*
miR-203a-3p	141	3,266	9,125	10,684	10,950	8,227	—
miR-203b-3p	203	6,067	15,822	13,313	11,540	8,187	—
miR-206-3p	574	5,480	25,693	33,058	35,970	48,114	*dapk3*; *c2cd4c*
miR-430a-3p	14,536	49,845	46,389	23,480	12,926	8,717	—
miR-430b-3p	1,247	17,167	13,004	6,534	3,060	2007	*ddx6*; *cplx2*
miR-430b-5p	564	171	173	84	56	35	—
miR-430c-3p	1,413	5,326	4,978	2,503	1,279	1,143	—

DS, developmental stage; abcc8, ATP binding cassette subfamily c member 8; alpk3, alpha kinase 3; c2cd4c, C2 calcium dependent domain containing 4c; cntnap2, contactin associated protein 2; cplx2, complexin 2; cyb5r4, cytochrome b5 reductase 4; dapk3, death associated protein kinase 3; ddx6, DEAD-box helicase 6; ddx56, DEAD-box helicase 56; eps15l1, epidermal growth factor receptor pathway substrate 15 like 1; gatad2b, GATA zinc finger domain containing 2b; ipo4, importin 4; ipo13, importin 13; kbtbd2, kelch repeat and BTB domain containing 2; mxra8, matrix remodeling associated 8; phka, phosphorylase kinase regulatory subunit alpha; pik3r4, phosphoinositide-3-kinase regulatory subunit 4; podxl, podocalyxin like; rbp4, retinol binding protein 4; tmem47, transmembrane protein 47.

### Differentially Expressed miRNAs, Expression Profiles

**FIGURE 3:**
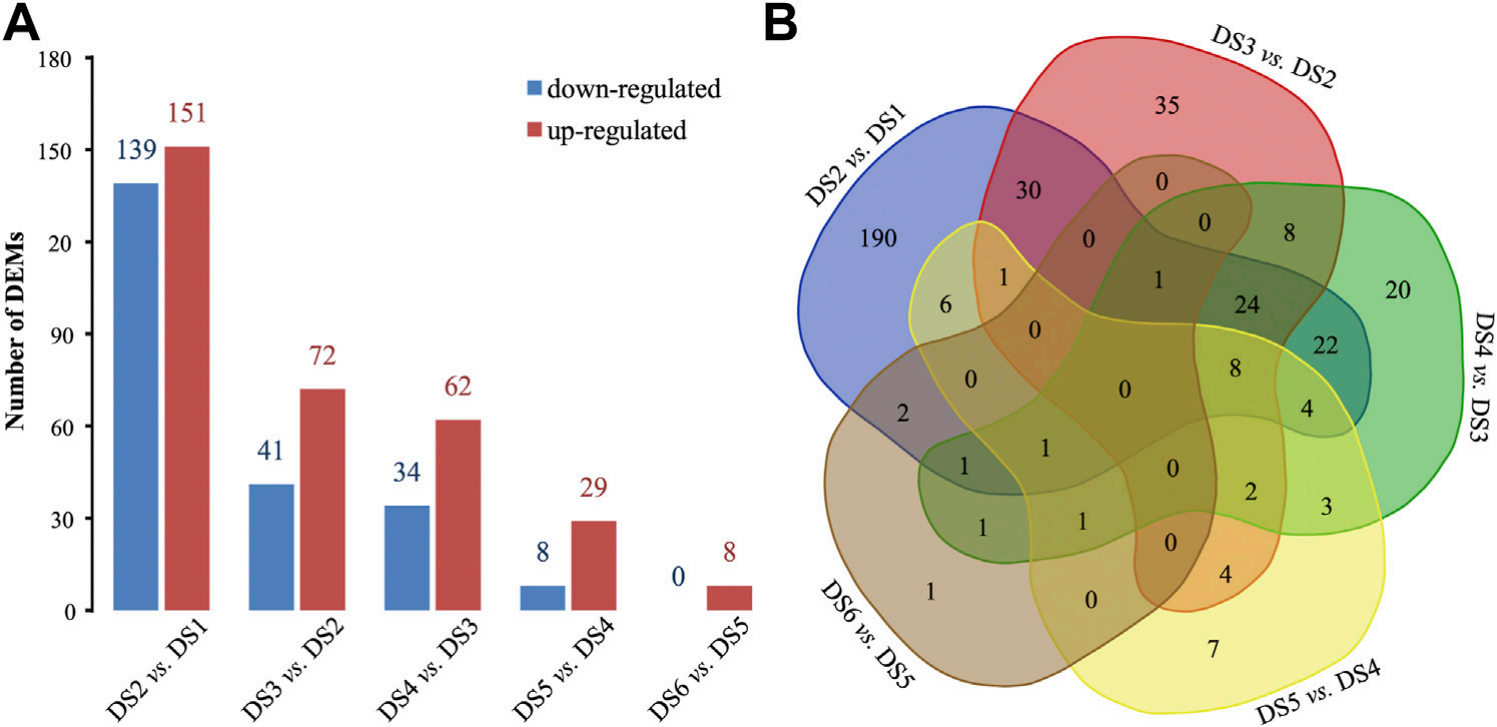
Pairwise comparisons to identify differentially expressed miRNAs (DEMs) between adjacent stages. **(A)** The bar plot statistic and **(B)** a Venn diagram.

A total of 372 and 240 pairwise DEMs were detected using different FC levels (2 or 4), and similar decreasing trends were detected for the DEMs identified using both FC levels (Supplementary Data Sheet S5). More DEMs were detected between the earlier adjacent stages, especially between DS2 and DS1, than between later stages, and more up-regulated DEMs were detected commonly in the pairwise comparisons (Figure 3A). Meanwhile, a certain proportion of DEMs were shared among the pairwise comparisons (Figure 3B). Three significant co-expression profiles of DEMs (*r* > 0.7, *p* < 0.05) were detected during the early development process, which showed continuously decreased expression (profile 8, containing 16 DEMs), continuously increased expression (profile 39, containing 116 DEMs), and obviously increased during the two early stages (profile 49, containing 90 DEMs) (Supplementary Figure S3).

### Target Prediction and Enrichment Analyses

Using sequence alignment and expression correlation analyses, a total of 1731 putative target transcripts were predicted to be negatively regulated (*r* < ‒0.80, *p* < 0.05) by 185 DEMs. The miRNAs could target one or several transcripts (range = 1–157), and some target transcripts were predicted to be regulated by several miRNAs (Supplementary Data Sheet S5). The predicted target genes of the most abundant miRNAs were listed in Table 1. Intriguingly, some DEMs showed alterative regulation in different periods, e.g., miR-27e significantly down-regulated and targeted *acaca*, *hkrp2*, and *nr3c1* genes during the period from DS1 to DS2, whereas the miR-27e significantly up-regulated and targeted other genes (*abtb2* and *ire-bp1*) during the period from DS2 to DS3. (Supplementary Figure S4).

**FIGURE 4:**
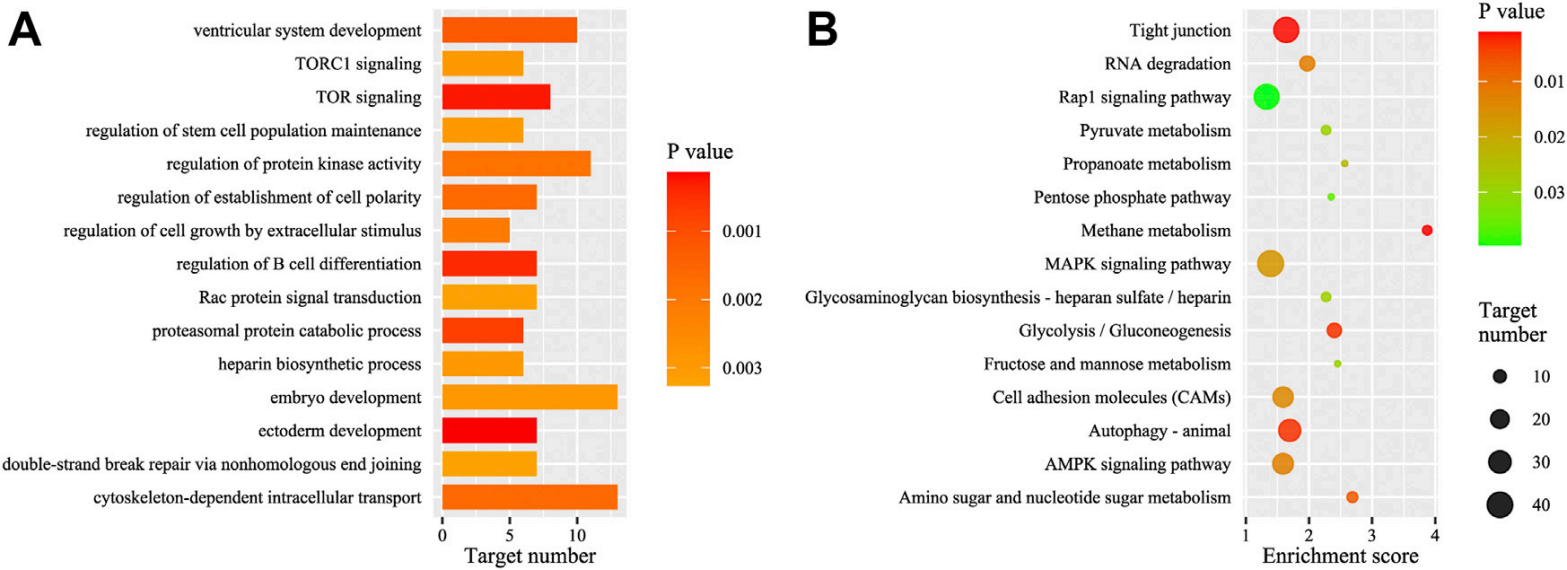
GO and KEGG analysis of the targets of the total differentially expressed miRNAs (DEMs). **(A)** The top GO biology process terms; **(B)** the top KEGG pathways. GO, gene ontology; KEGG, Kyoto Encyclopedia of Genes and Genomes.

**FIGURE 5:**
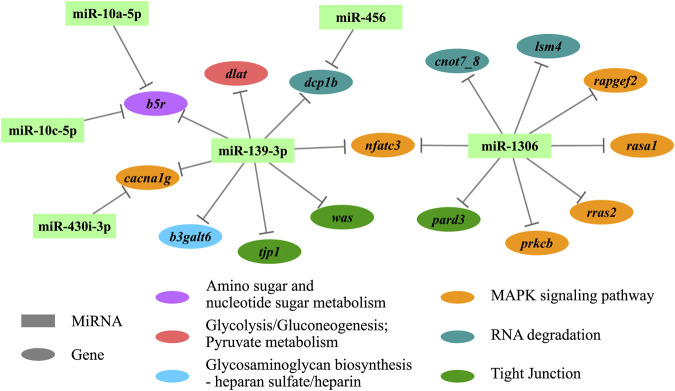
The hub network of differentially expressed miRNAs (DEMs) and their KEGG enriched target genes. KEGG, Kyoto Encyclopedia of Genes and Genomes.

Enrichment analyses (GO and KEGG) were performed based on the functional annotation of the target transcripts, and several biology processes (e.g., ectoderm development, embryo development, and ventricular system development) and pathways (e.g., autophagy—animal, RNA degradation, and tight junction) were enriched significantly (Figure 4). A miRNA-target gene hub network was constructed based on the DEMs and their KEGG enriched target genes (Figure 5), which also showed the multiple target relationships. In view of the obvious differences between the two earlier stages, the targets of DEMs defined with a stricter threshold (FC > 4) were used for enrichment analyses. Many important biology processes and pathways were enriched corresponding to the DEMs from the DS2 *vs* DS1 comparison, including development, differentiation, growth, and signaling related terms (Supplementary Data Sheet S4, and Supplementary Figure S5). Notably, some KEGG pathways seem to be related to maternal genome expression (e.g., aldosterone synthesis and secretion, GnRH signaling pathway, and ovarian steroidogenesis).

**FIGURE 6:**
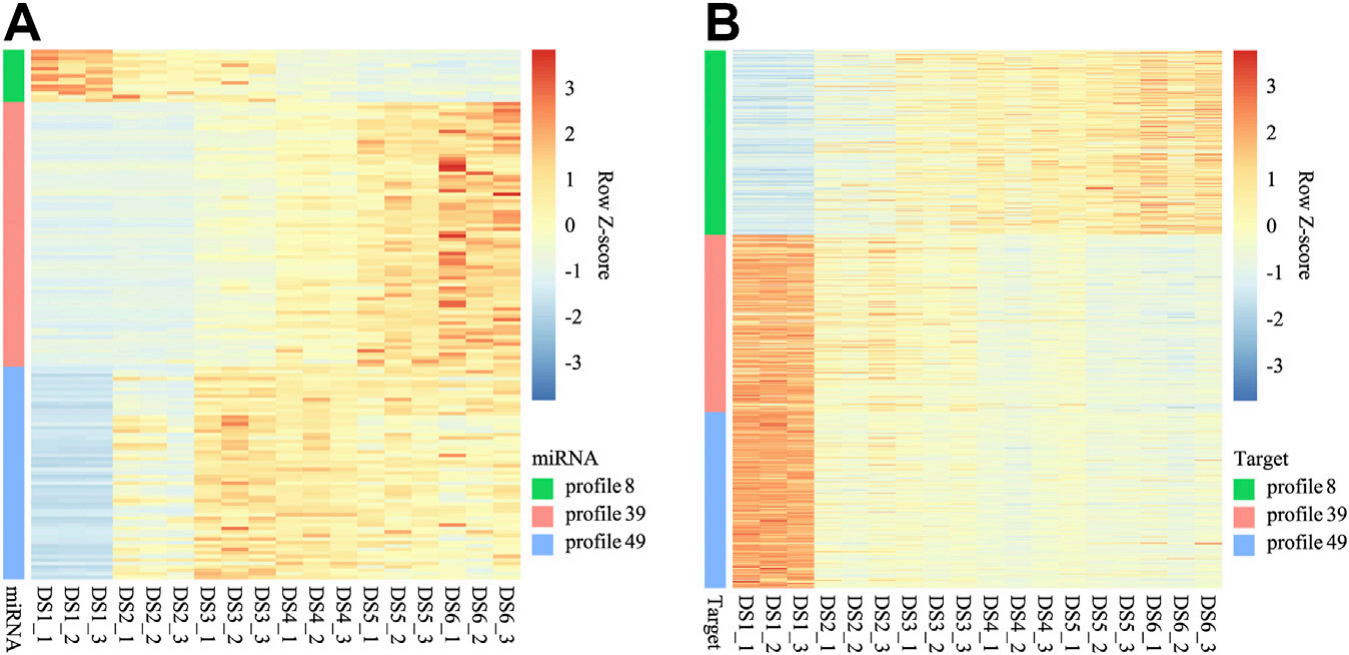
Heat maps for expression of miRNAs **(A)** of three co-expression profiles and their targets **(B)**.

Base on the correlation analyses conducted between the DEMs and the expression of their targets expressions, a broadly negative regulation was detected for three expression profiles of DEMs (Figure 6), and the corresponding enrichment revealed that many biology processes and pathways were enriched within certain expression profiles (Supplementary Data Sheet S5).

### Quantitative Real-Time-PCR Validation for Differentially Expressed miRNAs and Their Targets

**FIGURE 7:**
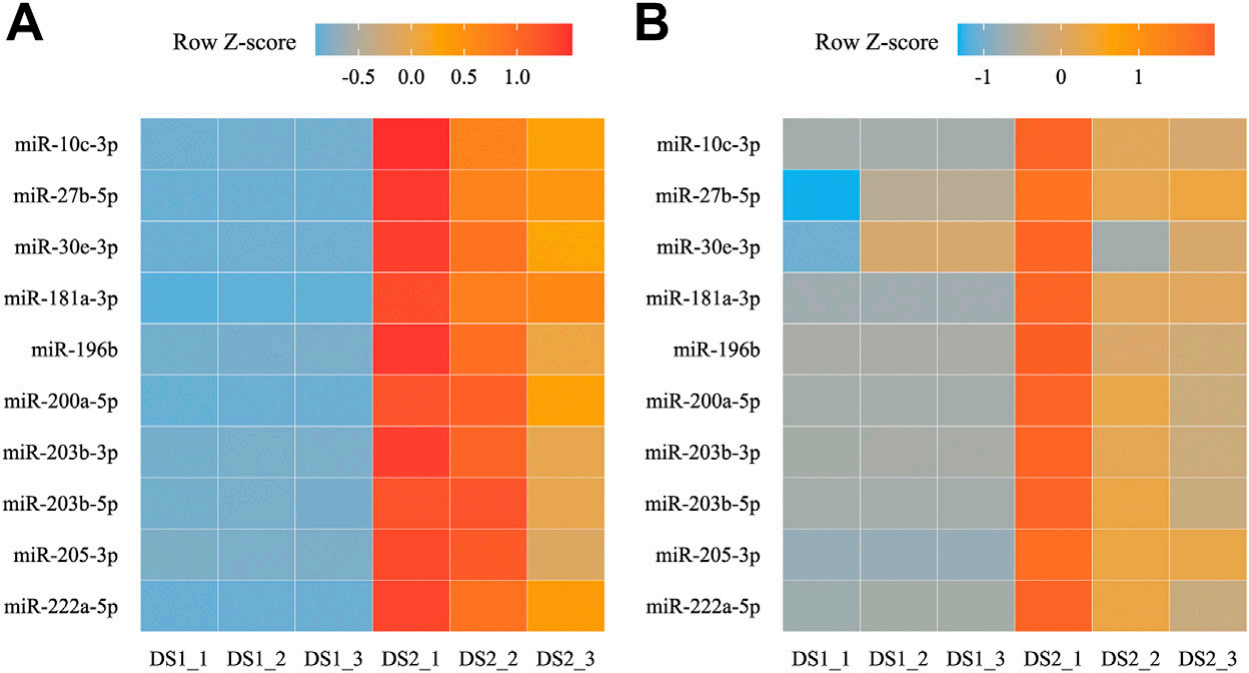
Heat maps for transcripts per million (TPM) **(A)** and relative expression **(B)** of ten differentially expressed miRNAs (DEMs) between two early developmental stages.

Among the combined the DEMs and expression profile results, ten up-regulated miRNAs and their targets (five transcripts) in the DS2 *vs* DS1 comparison were examined using qRT-PCR to validate the RNA-seq data and analyses. The generally expression trends between the RNA-seq and qRT-PCR data were consistent, which revealed by the Z-score normalization for each miRNA (Figure 7). In detail, the relative expression of all the DEMs showed an up-regulated trend from DS1 to DS2 (the FC ranged from 1.39 to 453.11). Among them, the relative expressions of nine miRNAs showed significant different between the pairwise comparison (*p* < 0.05, Wilcoxon test), and significantly positive correlation (*r* > 0.85, *p* < 0.05) was detected between the RNA-seq data and the qRT-PCR data for each miRNA of nine DEMs (Supplementary Data Sheet S1). Additionally, all five miRNA targets were detected with down-regulated trends from DS1 to DS2 (the FC ranged from 0.008 to 0.159), and showed with significant different between pairwise comparison (*p* < 0.05, T test or Wilcoxon test) (Supplementary Data Sheet S1).

## Discussion

In the present study, in the earliest stage of bighead carp development (DS1), a peak at around 22 nt representing miRNAs and another distinct peak around 26–28 nt representing longer piRNAs (Piwi-interacting RNAs) were detected. Studies have demonstrated that piRNAs are essential for gene silencing and transposon regulation during germ cell differentiation and gonadal development (Khurana and Theurkauf, 2010; Grentzinger et al., 2012). In teleost species, piRNAs are mainly expressed in ovaries and testes (Houwing et al., 2007; Wang et al., 2017). Moreover, most piRNAs are maternally deposited and are degraded gradually as development proceeds (Wei et al., 2012; Yao et al., 2014). However, evidence of the regulation of piRNAs during early development is limited. In contrast to piRNAs, many miRNAs show temporal expression patterns, such as those expressed during MZT and metamorphosis (Bizuayehu and Babiak, 2014; Herkenhoff et al., 2018). At the global level, the dynamically increasing trend of miRNA expression, and the obvious separation among samples from different stages revealed by PCA, as well as the homogeneity of miRNA expression among samples from each stage detected in this study, indicated the close involvement of miRNAs in the regulation of early developmental transitions of bighead carp.

In the present study, the sequencing data showed that miRNA expression increased during the early development process. However, a small proportion of miRNAs were highly abundant during the early development stages (52 miRNAs’ TPM ≥1,000), especially at the late organogenesis stages. The low expression of almost all miRNAs in the early stages, which became increasingly diverse and complex as development proceeded, was reported in zebrafish (Chen et al., 2005; Thatcher et al., 2007; Wei et al., 2012; Kaitetzidou et al., 2015). We observed that several miRNAs (e.g., miR-430a-3p, miR-430b-3p, and miR-430c-3p) that were increasingly expressed at the earlier stages and then decreased in the later stages belonged to the miR-430 family, which is involved in clearance of maternal transcripts and embryonic morphogenesis (Giraldez et al., 2006; Thatcher et al., 2007). Similarly, high miR-430 expression was observed in the early development of zebrafish (Wei et al., 2012), common carp (Wang et al., 2017; Wang et al., 2021) and Atlantic halibut (*Hippoglossus hippoglossus*) (Bizuayehu et al., 2012). In Atlantic halibut, miRNA-430 expression ceased after hatching (Bizuayehu et al., 2012), whereas it was expressed for a longer period according to the results of this study and in common carp (Wang et al., 2017; Wang et al., 2021). This pattern of delayed expression was also observed for many miRNAs associated with early development (Ason et al., 2006). In particular, miR-430 also has important functions in the metamorphosis of *Paralichthys olivaceus* early development (Li et al., 2020), and during ovary differentiation of common carp (Wang et al., 2017). Although, the miRNAs shared same seed sequence, the different expression patterns and special functions of the miR-430 family were detected in different species. Which indicated that conservation of miRNAs did not reflect conservation of their expression among species (Ason et al., 2006). miRNAs of several families, including miR-1, miR-21, miR-22, and miR-26, were also commonly detected in early development stages of other fishes (Chen et al., 2005; Wang et al., 2017; Sarropoulou et al., 2019; Wang et al., 2021). Among them, miR-21 expression in teleost species is involved in development (Chen et al., 2005), maternal transcript degradation (Ramachandra et al., 2008), and stress responses (Zhao et al., 2016). The high expression levels of miR-1 and miR-206, which are muscle-specific miRNAs, were observed in the early stages of medaka and zebrafish development (Ason et al., 2006), and during zebrafish gastrulation, miR-206 was identified as essential for the regulation of cell movement (Liu et al., 2012). Overall, the identified highly expressed miRNAs might function in various regulatory pathways in early development, which will require further investigation.

The large proportion of miRNAs showing low expression levels might not be critical for the early developmental processes in bighead carp, which was also found in other teleost species (Bizuayehu et al., 2012; Yao et al., 2014; Kaitetzidou et al., 2015; Sarropoulou et al., 2019). Although some miRNAs were expressed at a low level and/or showed high cellular specificity (Wienholds et al., 2005), we cannot rule out the possibility that some of them might be highly and widely expressed only at certain developmental stages (Alberti and Cochella, 2017). In this study, some miRNAs (let-7, miR-122, and miR-192) showed moderate expression and their levels increased gradually during the development process, reaching a high level in the later stages (Chi et al., 2011; Bizuayehu et al., 2012). Among them, the let-7 family is related to tissue proliferation, growth, and metamorphosis in teleosts (Kloosterman et al., 2004; Ason et al., 2006; Bizuayehu et al., 2012); however, let-7 is also present at moderate level in the early development stages of zebrafish (Wei et al., 2012). Actually, many miRNAs may play roles at the periphery of development, lower expressed miRNAs are likely to be expressed at later time points in development and in a cell type-restricted manner (Alberti and Cochella, 2017). For example, specific mRNAs (miR-24 or miR-139 families) are known to limit phenotypic variation in zebrafish embryos (Kasper et al., 2017), and these miRNAs showed lower expression levels in the present study.

Interestingly, the highly expressed miRNAs identified in this study were almost all known (or conserved) miRNAs. It is not surprising that many conserved animal miRNAs are abundantly involved in core developmental process (Chen and Rajewsky, 2007; Alberti and Cochella, 2017), which might involve the maintenance of basic cellular activities. By contrast, most of the novel miRNAs showed lower expression in this study, which is in accordance with other reports (Chi et al., 2011; Robledo et al., 2017; Wang et al., 2017). It was hypothesized that gene expression canalization involves miRNAs, which were linked to species-specific evolution processes (Chen and Rajewsky, 2007; Wu et al., 2009; Lee et al., 2010).

A dynamic decrease was revealed among the DEMs in the pairwise comparisons, with more DEMs being identified in the earlier developmental stages, which coincided with the dynamic changes in transcripts expression in the early development of bighead carp (Fu et al., 2019). MiRNAs implement their biology functions through targeting transcripts and suppressing gene expression at the posttranscriptional level. Therefore, we further investigated the possible association of miRNAs with genes by performing target prediction and expression correlation analysis. Complex interactions were detected in the miRNA-gene network, i.e., one miRNA can target more than one gene, and different miRNAs could also target the same gene. Meanwhile, the genes might play roles in different biology processes and pathways, or one gene might play multiple roles. Similar results were reported in other studies (Robledo et al., 2017; Wang et al., 2021), indicating that some miRNAs might be involved in a wide range of gene regulations in various biological processes. These observations support the concept of functional redundancy among miRNAs (Alberti and Cochella, 2017). The cooperation of miRNAs (miR-35 and miR-58) by targeting the same gene has been illustrated in animal embryonic development (Sherrard et al., 2017). In this study, we observed that some miRNAs were identified as members of families (let-7, miR-1, miR-430) in which multiple members share the same seed sequence; however, some homologs had different expression levels or patterns, and were also predicted to have different target genes in the development process. In the present study, the same or different target genes with the same or different expression trends (up- or down-regulated) were detected for single DEM at different periods of the development process, which has been less reported. The complex (or dynamic) gene regulation of miRNAs speculated in this study might be associated with the specific spatiotemporal patterns of miRNA expression (Alberti and Cochella, 2017), as well as influence of the variation of miRNAs and target binding sites (Nepal et al., 2013; Kasper et al., 2017; Shi et al., 2020). In addition, many miRNAs were co-expressed and exerted a broad negative regulation on target genes during early bighead carp development. Similar co-expression of miRNAs has been detected commonly in animal development (Wang et al., 2017; Do et al., 2019; Chen et al., 2020), further indicating the roles of miRNAs in providing developmental robustness.

The miRNAs play roles via suppression targets’ expression, a widely negatively correlation between expressions of miRNAs and their targets was revealed in this study. Overall, enrichment analyses were conducted based on the functional annotation of the DEMs’ target transcripts, leading to functional characterization of miRNAs’ regulation in early development. Many development-relative terms in GO biology process were enriched for DEMs’ target transcripts, including ectoderm development, embryo development, and ventricular system development. The KEGG analysis identified several enriched pathways might be associated with the development process, e.g., AMPK signaling, MAPK signaling, RNA degradation, and tight junction pathways. The MAPK signaling pathway has also been associated with miRNA-target regulation in animal development (Kaitetzidou et al., 2015; Wang et al., 2017; Jing et al., 2021). Moreover, several sex hormone-related pathways were enriched for DEMs’ target transcripts in the earliest pairwise comparison (DS2 *vs* DS1), including aldosterone synthesis and secretion, GnRH signaling, and ovarian steroidogenesis pathways. The results provided more evidence for the roles of miRNAs in the degradation of maternal transcripts. In view to the certain miRNA’s biological functions, the pairwise miRNA-target relationship might to be better used for evaluation. For example, the miR-430b-3p was predictively targeted to *ddx6* gene, which encodes a member of the DEAD box protein family (aka. Rck/P54). It plays a role in mRNA decapping in the process of mRNA degradation (Fenger-Grøn et al., 2005), which may be required for miRNA-induced gene silencing.

In conclusion, the miRNA repertoire and expression characteristics were revealed in this study, which provided a first insight into the involvement of miRNAs during the early development of bighead carp. Additionally, the high consistency of the RNA-seq and qRT-PCR data, illustrated the reliability of the sequencing and analysis protocols. We observed that miRNAs effected dynamic and wide ranging regulation of genes during the development processes, which might contribute to a better understanding of the molecular dynamic changes and regulations during fish embryogenesis and development.

## Data Availability

The original contributions presented in the study are publicly available. This data can be found here: National Center for Biotechnology Information (NCBI) BioProject database under accession number PRJNA781113.
