# Supplementary material for: Dynamic Expression and Gene Regulation of MicroRNAs During Bighead Carp (Hypophthalmichthys nobilis) Early Development
Source: Front Genet. 2022 Jan 19;12:821403. doi: 10.3389/fgene.2021.821403 (PMC8809360; doi:10.3389/fgene.2021.821403)
Supplement: Supplementary file 3 [file Image6.PDF]

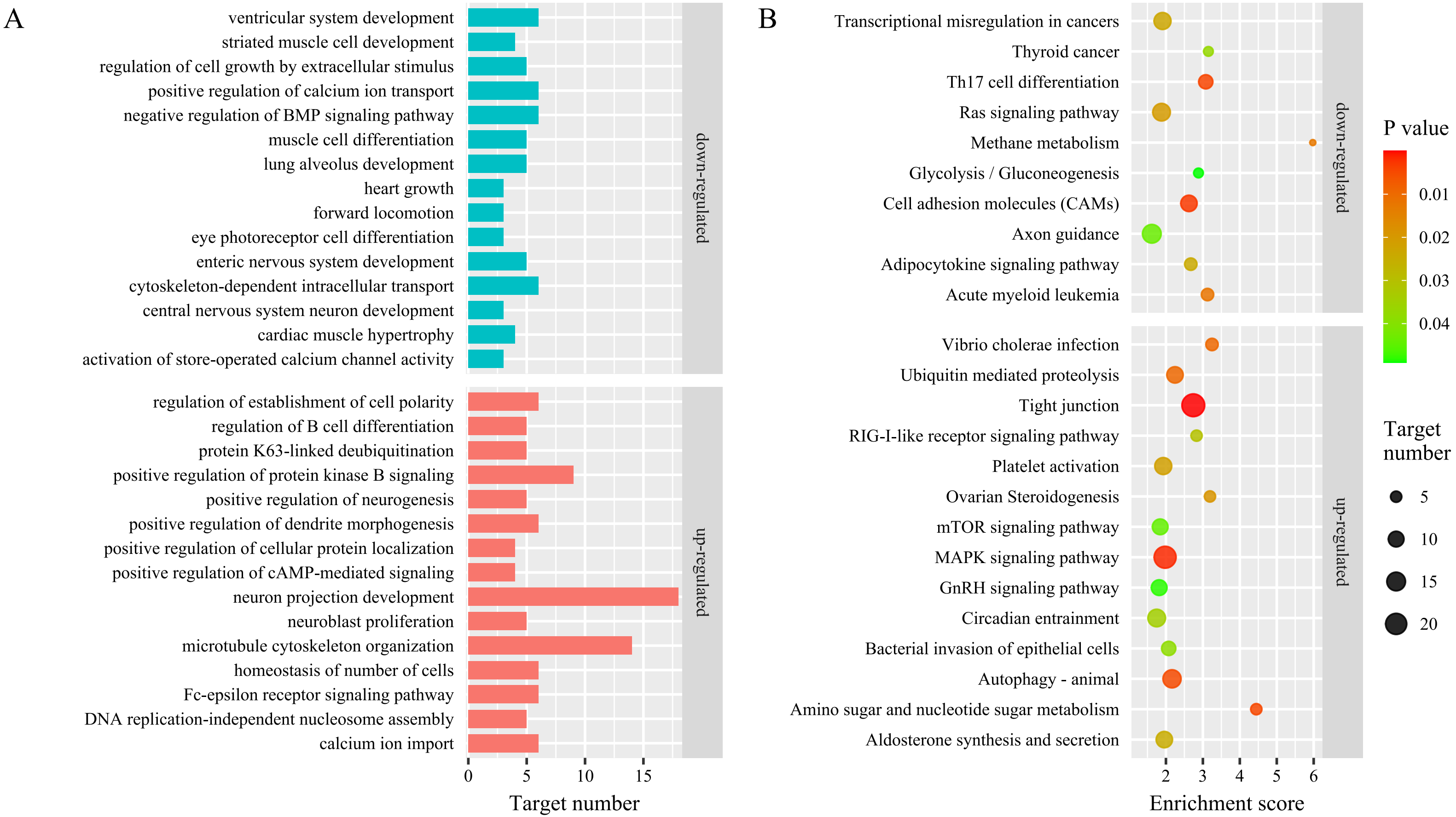

Supplementary Figure 6. GO and KEGG analysis of the targets of differentially expressed miRNAs (DEMs) from the DS2 *vs.* DS1 comparison. **(A)** The top GO biology process terms; **(B)** the top KEGG pathways. GO, gene ontology; KEGG, Kyoto Encyclopedia of Genes and Genomes.
