# Supplementary material for: Dynamic Expression and Gene Regulation of MicroRNAs During Bighead Carp (Hypophthalmichthys nobilis) Early Development
Source: Front Genet. 2022 Jan 19;12:821403. doi: 10.3389/fgene.2021.821403 (PMC8809360; doi:10.3389/fgene.2021.821403)
Supplement: Supplementary file 4 [file Image4.pdf]

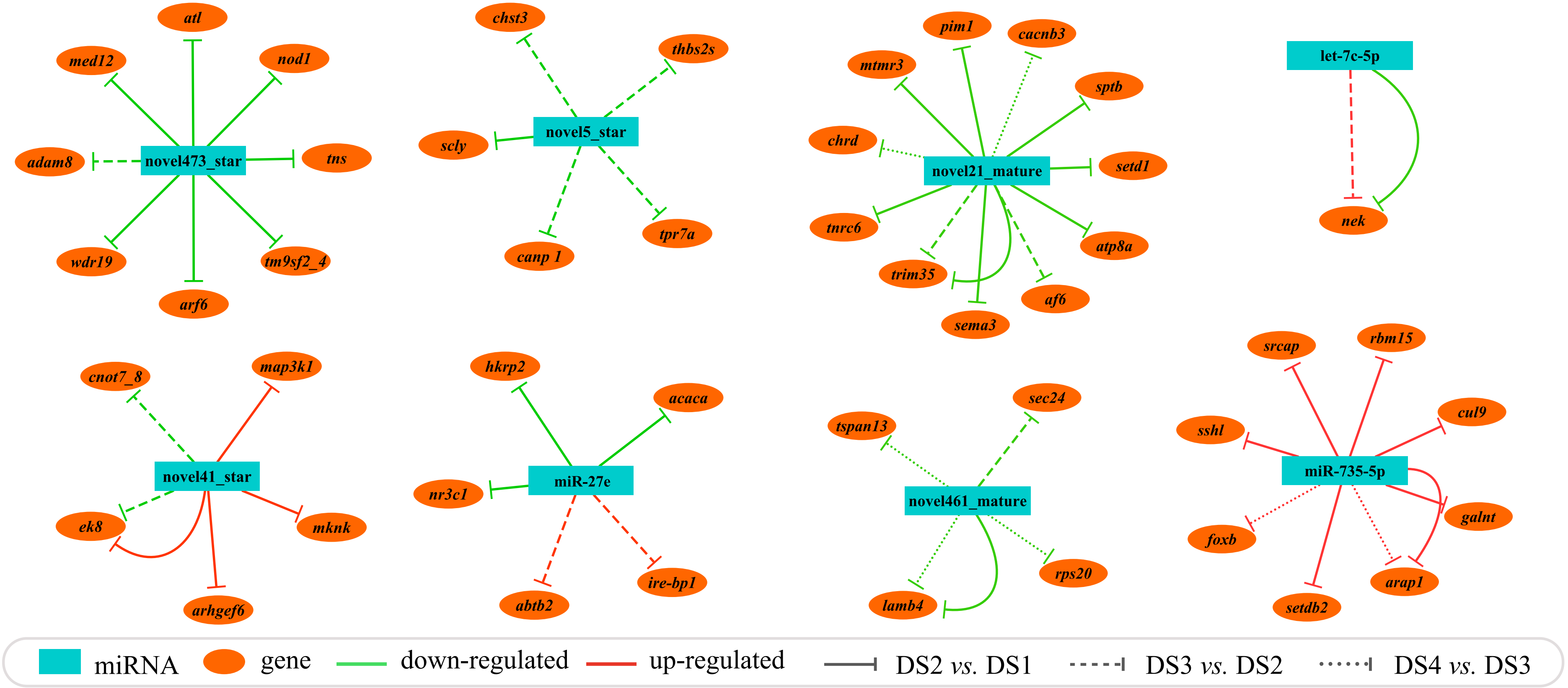

Supplementary Figure S4. The intersection of differentially expressed miRNAs (DEMs) and their target genes network between pairwise comparisons.
