# Supplementary material for: Dynamic Expression and Gene Regulation of MicroRNAs During Bighead Carp (Hypophthalmichthys nobilis) Early Development
Source: Front Genet. 2022 Jan 19;12:821403. doi: 10.3389/fgene.2021.821403 (PMC8809360; doi:10.3389/fgene.2021.821403)
Supplement: Supplementary file 5 [file Image2.pdf]

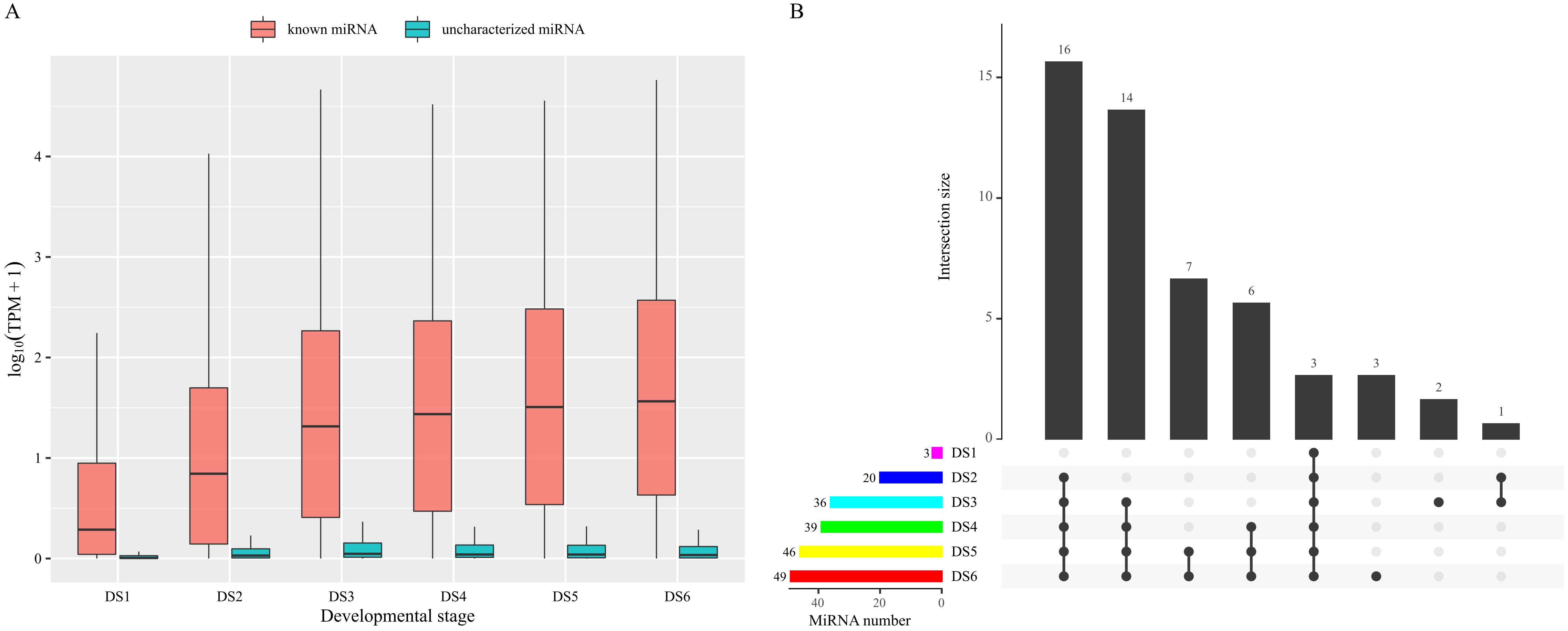

Supplementary Figure S2. Box plot for known and uncharacterized miRNA expression in each stage **(A)**, and the intersection of abundant miRNA (transcripts per million, TPM $\geq$ 1000) numbers among stages **(B)**.
