# Supplementary material for: Dynamic Expression and Gene Regulation of MicroRNAs During Bighead Carp (Hypophthalmichthys nobilis) Early Development
Source: Front Genet. 2022 Jan 19;12:821403. doi: 10.3389/fgene.2021.821403 (PMC8809360; doi:10.3389/fgene.2021.821403)
Supplement: Supplementary file 6 [file Image3.pdf]

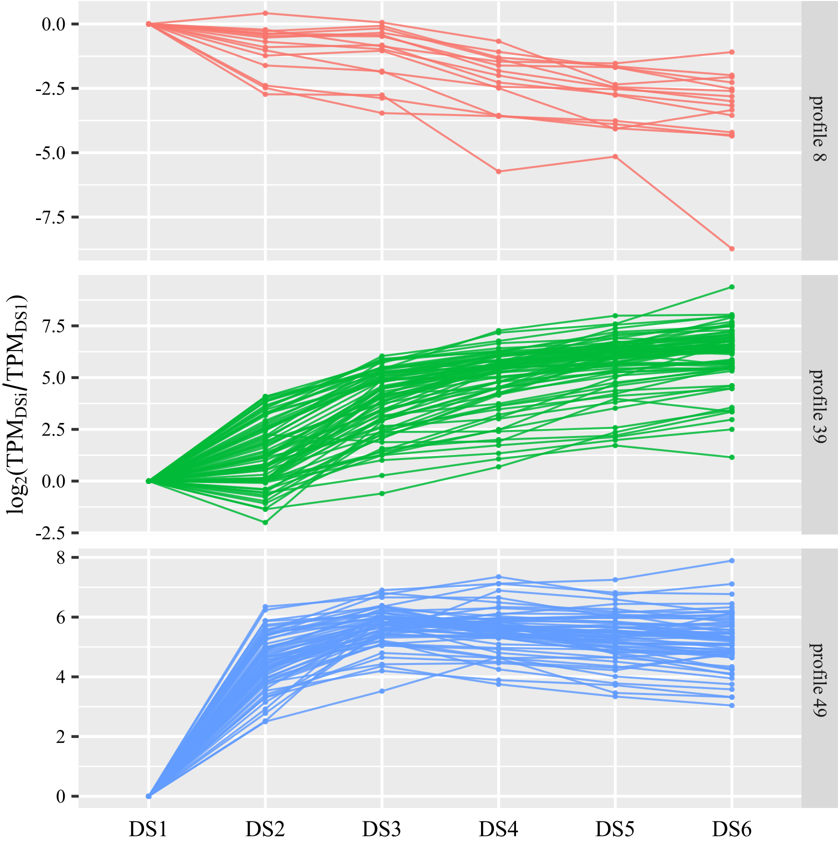

Supplementary Figure S3. Line plot of three co-expression profiles of differentially expressed miRNAs (DEMs).
