# Supplementary material for: Dynamic Expression and Gene Regulation of MicroRNAs During Bighead Carp (Hypophthalmichthys nobilis) Early Development
Source: Front Genet. 2022 Jan 19;12:821403. doi: 10.3389/fgene.2021.821403 (PMC8809360; doi:10.3389/fgene.2021.821403)
Supplement: Supplementary file 9 [file Image1.pdf]

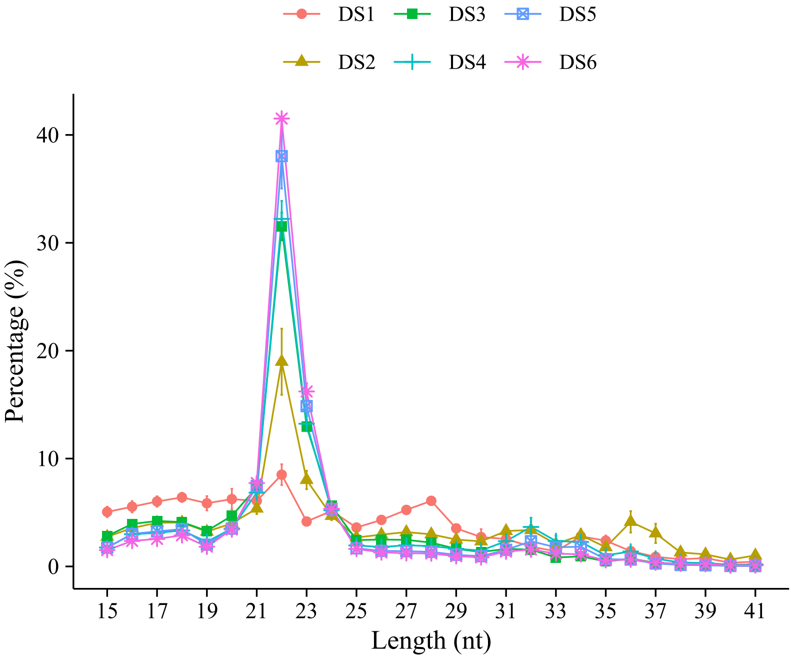

Supplementary Figure S1. The distribution of the lengths of the small RNAs in the sequencing data
